# The Dynamical Behaviors for a Class of Immunogenic Tumor Model with Delay

**DOI:** 10.1155/2017/1642976

**Published:** 2017-10-25

**Authors:** Ping Bi, Zijian Liu, Mutei Damaris Muthoni, Jianhua Pang

**Affiliations:** ^1^Department of Mathematics, Shanghai Key Laboratory of PMMP, East China Normal University, 500 Dongchuan Rd., Shanghai 200241, China; ^2^College of Mathematics and Statistics, Chongqing Jiaotong University, Chongqing 400074, China; ^3^School of Science, Guangxi University of Science and Technology, Liuzhou 545006, China

## Abstract

This paper aims at studying the model proposed by Kuznetsov and Taylor in 1994. Inspired by Mayer et al., time delay is introduced in the general model. The dynamic behaviors of this model are studied, which include the existence and stability of the equilibria and Hopf bifurcation of the model with discrete delays. The properties of the bifurcated periodic solutions are studied by using the normal form on the center manifold. Numerical examples and simulations are given to illustrate the bifurcation analysis and the obtained results.

## 1. Introduction

For the longest time, malignant tumors have posed a threat to human lives. Effective strategies based on the immune system have been championed to complement traditional methods of cancer treatment, such as surgery, radiation, and chemotherapy. Cancer immunotherapy is the use of the immune system to treat cancer. Immunotherapy is used to provoke the immune system into attacking the tumor cells by using these cancer antigens as targets. Cell types that can be used in this way are natural killer cells, lymphokine-activated killer cells, cytotoxic T cells, and dendritic cells. There has been much interest in mathematical models describing the interaction between tumor cells and effector cells of the immune system; see [1–6]. An ideal model can provide insight into the dynamics of interactions of the immune response with the tumor and may play an important role in understanding of cancer and developing effective drug therapies. Developing ideal models for such complex processes is not easy. Simple models which display some useful immunological phenomena have been proposed and studied extensively. See Bi and Xiao [7], Galach [4], and Yafia [8–10], and the references cited therein. In 1994, Kuznetsov et al. [11] introduced a model, which describes competition between the tumor and immune cells. It also describes the response of effector cells (ECs) to the growth of tumor cells (TCs). It is assumed that the tumor cells are “immunogenic” and thus subject to immune attack by cytotoxic (CT) or natural killer (NK) cells. This model studies the infiltration of TCs by ECs and also the possibility of ECs inactivation.* It is assumed that interactions between ECs and TCs in vitro can be described by the following kinetic scheme describing interactions between ECs and TCs:*

(1) E+T⇄nnk−1nnnnk1nnC⟶k2E∗+T⟶k3T∗+E

where *E*, *T*, *C*, *T*^*∗*^, *E*^*∗*^ are the local concentrations of *EC*s, *TC*s, *EC*-*TC* complexes, inactivated ECs, and TCs, respectively. The parameters *k*_1_, *k*_−1_, *k*_2_ and *k*_3_ are nonnegative constants, which describe the conversion rates of differential cells. Then Kuznetsov and Taylor's model is as follows:

(2) dEdt=s+FC,T−d1E−k1ET+k−1+k3C,dTdt=aT1−bTtot−k1ET+k−1+k2C,dCdt=k1ET−k−1+k2+k3C,dT∗dt=k3C−d2T∗,dE∗dt=k2C−d3E∗,

where *C* is the normal rate of the flow of adult *EC*s into the tumor site, *F*(*C*, *T*) describes the accumulation of effector cells in the tumor cells localization due to the presence of the tumor. *F*(*C*, *T*) = *fC*/(*g* + *T*), (*f*, *g* are constants). If *T*_tot_ ≈ *T*  (*T*_tot_ = *T* + *C*), it is reasonable to assume *dC*/*dt* ≈ 0, that is, *C* ≈ *kET*  (*k* = *k*_1_/(*k*_−1_ + *k*_2_ + *k*_3_)), then *F*(*C*, *T*) = *F*(*E*, *T*). Then we only need to analyze the dynamical behavior of the first two equations.

In 2003, Galach [4] suggested that the function *F* is in the Michaelis-Menten form *F*(*E*, *T*) = *θET*, and thus (2) can be simplified as

(3) dxdt=c+θxy−m1xy−d1x,dydt=ay1−by−m2xy,

where *x* is the local concentrations of *EC*s, and *y* is the local concentrations of *TC*s, *m*_1_ = *kk*_2_, *m*_2_ = *kk*_3_, and all the parameters are positive. The properties of the model (3) is studied in [7–9]. The dynamical behaviors and the bifurcation of the model with delay are also studied by [7, 10].

In this paper, we analyze the dynamics of an immune response function with Michaelis-Menten form *F*(*E*, *T*) = *pET*/(*g* + *T*), where *p* and *g* are positive constants, that is,

(4) dEdt=s+pETg+T−mET−dE,dTdt=aT1−bT−nET.

In order to simplify the original model, we nondimensionalize (4) by choosing scale for *E* and *T* cell population, respectively. Let *τ* = *nT*_0_*t*, and replace *τ* with *t*; thus the model can be written as follows:

(5) dxdt=σ+αxy1+y−μxy−δx,dydt=fy1−βy−xy,

where *y* = *T*/*g*, *x* = *E*/*g*, *τ* = *tng*, *σ* = *s*/*ng*^2^, *α* = *p*/*ng*, *μ* = *m*/*n*, *δ* = *d*/*ng*, *f* = *a*/*ng*, *β* = *bg* and all the above parameters are positive.

This paper is organized as follows: In Section 2, the model without delay is considered, and the conditions for existence and stability of equilibria are given. In Section 3, the model with delay is studied. The existence of Hopf bifurcation is obtained; the direction and stability of bifurcated periodic solutions are also given with the help of center manifold and bifurcation theories. At the end of this paper, numerical results are given to illustrate the main results of this paper.

## 2. Existence and Stability of the Equilibria

It is easy to see that (5) has a tumor-free equilibrium *P*_0_ = (*σ*/*δ*, 0). In order to find the positive equilibria *P*_1_ = (*x*^*∗*^, *y*^*∗*^), we need to solve the following equations:

(6) dxdt=σ+αxy1+y−μxy−δx=0,dydt=fy1−βy−xy=0.

Equation (6) is reduced to the following cubic polynomial:

(7) $$\begin{array}{c} a_{1}y^{3}+a_{2}y^{2}+a_{3}y+a_{4}=0, \end{array}$$

where *a*_1_ = *fβμ*, *a*_2_ = *f*(−*αβ* + *βδ* − *μ* + *βμ*), *a*_3_ = *fα* − *fδ* + *fβδ* − *fμ* + *σ*, *a*_4_ = −*fδ* + *σ*. Obviously, system (5) in *R*_2_^+^ is as “well behaved” just as in biology. We have the following lemma by qualitative analysis.

**Lemma 1 .**

If *μ* > *α*, then the solutions of (5) are invariable in *D*:

(8) $$\begin{array}{c} D=\left\{\;\left(\;x,y\;\right)\;|\;0\leq x\leq \frac{2\sigma }{\delta },\;\;0\leq y\leq \frac{2}{\beta }\;\right\}. \end{array}$$

**Proof**

If *x* = 0 and 0 < *y* < 2/*β*, then

(9) dxdtx=0=σ>0,dydtx=0=fy1−βy>0for  0<y<1β,<0for  1β<y<2β.

Similarly, if *y* = 0 and 0 < *x* < 2*σ*/*δ*, one has

(10) dxdty=0=σ−δx>0,for  0<x<σδ,<0,for  σδ<x<2σδ,dydty=0=0.

Also, if *y* = 2/*β* and 0 < *x* < 2*σ*/*δ*, we have

(11) dydty=2/β, 0<x<2σ/δ=yf1−βy−xy=2/β, 0<x<2σ/δ<0,dxdty=2/β, 0<x<2σ/δ=σ+x2αβ−β+22μ+δβββ+2y=2/β, 0<x<2σ/δ<0,

and, then, there exists a positive number *x*^*∗*^, which is given by

(12) $$\begin{array}{c} x^{*}=\frac{\sigma \beta \left(\beta +2\right)}{\left(\beta +2\right)\left(2\mu +\delta \beta \right)-2\alpha \beta }. \end{array}$$

Such that *dx*/*dt* < 0 when *x* > *x*^*∗*^. Similarly, *dx*/*dt* > 0 when *x* < *x*^*∗*^. On the other hand, one has

(13) $$\begin{array}{c} 0<\frac{\sigma \beta \left(\beta +2\right)}{\left(\beta +2\right)\left(2\mu +\delta \beta \right)-2\alpha \beta }<\frac{2\sigma }{\delta } \end{array}$$

with the help of *μ* > *α*. That is 0 < *x*^*∗*^ < 2*σ*/*δ*.

When *x* = 2*σ*/*δ* and 0 < *y* < 2/*β*, we can prove the result in a similar way. This finishes the proof.

It is easy to see that (5) has one tumor-free equilibrium *P*_0_ = (*σ*/*δ*, 0). With the distribution of the eigenvalues, we can easily obtain the following results.

**Theorem 2 .**

If *f* > *σ*/*δ*, then *P*_0_ is unstable. If *f* = *σ*/*δ*, *P*_0_ is stable. If *f* < *σ*/*δ*, *P*_0_ is asymptotically stable.

**Theorem 3 .**

If >*fδ*, *δβ* > *μ* > *α*, then *P*_0_ is globally asymptotically stable.

**Proof**

When >*δf*, *βδ* > *μ* > *α*, then it is easy to see that

(14) a1>0,a4=−fδ+σ>0,a3=fα+σ−fδ+fβδ−μ>0,a2=fβμ−α+βδ−μ>0.

This implies that *P*_0_ is the only positive equilibrium.

Noting *σ* > *df*, we know that *P*_0_ is locally stable with the help of Theorem 2. In the remaining part of the proof, we only need to prove the global stability. By Lemma 1, we know that the following domain *D* is invariable:

(15) $$\begin{array}{c} D=\left\{\;\left(\;x,y\;\right)\;|\;0\leq x\leq \frac{2\sigma }{\delta },\;\;0\leq y\leq \frac{2}{\beta }\;\right\}. \end{array}$$

Then it is easy to prove that *dx*/*dt* < 0 as *x* > 2*σ*/*δ*. In addition, when *y*_0_ > 2/*β*,

(16) $$\begin{array}{c} \frac{dy}{dt}=fy\left(\;1-\beta y-x\;\right)=-fy\left(\;\left(\;1-\beta y\;\right)+xy\;\right)<0. \end{array}$$

This shows that the vector fields are moving towards *D* as *t* increases.

Let Dulac function *B* = 1/*y*. Then

(17) $$\begin{array}{c} \frac{\partial \left(BP\right)}{\partial x}+\frac{\partial \left(BQ\right)}{\partial y}\leq \alpha -\mu -f\beta <0. \end{array}$$

Hence there are no closed trajectory surrounds *P*_0_ in field *D*. That is, the result follows.

Using the original parameters, we can give the results as follows.

**Theorem 4 .**

If *s* > *da*/*n*, *db* > *m* > *p*/*g*, system (5) has only one critical equilibrium *P*_0_ = (*σ*/*δ*, 0). Furthermore, *P*_0_ is globally asymptotically stable.

**Remark 5 .**

Theorem 4 is an instructive results to kill the tumor cells. The tumor cells will be killed out by the immune cells sooner or later under the above conditions; then we only need to take the necessary measures to control the parameters to satisfy the inequity in Theorem 4.

In the following, we will study the existence and stability of the tumor-present equilibrium *P*_1_(*x*^*∗*^, *y*^*∗*^). Similar to the proof of Lemma  2.4 in [12], we can easily obtain the following results.

**Theorem 6 .**

For the number of positive equilibria, we can get the following results:

1. If >*δ* > *μ*/*β* > *μ*, *σ* < *fδ*, and Δ < 0, then (5) has three distinct positive roots.
2. If one of the following conditions is satisfied, then (5) has two distinct positive roots:
  - *α* > *δ* > *μ*/*β* > *μ*, *σ* > *fδ*, and Δ < 0.
  - *α* > *δ* > *μ*/*β* > *μ*, *σ* > *fδ* and Δ = 0.
3. Assume one of the following conditions is satisfied, then (5) has one positive root:
  - *α* > *δ* > *μ*/*β* > *μ*, *σ* < *fδ*, and Δ < 0.
  - *α* > *δ* > *μ*/*β* > *μ*, *σ* < *fδ*, and *A* = *B* = 0.
  - *δ* > *α*, *β* > 1, *α* + *βδ* < *μ*, *σ* < *δf*, and Δ > 0.
  - *α* > *δ* > *μ*/*β* > *μ*, *σ* < *fδ*, and Δ = 0.

The corresponding Jacobian matrix at *P*_1_(*x*^*∗*^, *y*^*∗*^) is

(18) $$\begin{array}{c} J=\left(\begin{array}{cc} \frac{-\sigma }{x^{*}} & \frac{\alpha x^{*}}{{\left(1+y^{*}\right)}^{2}}-\mu x^{*}\\ -y^{*} & -f\beta y^{*} \end{array}\right). \end{array}$$

Thus, we can give the following results.

**Theorem 7 .**

If *σβ* > *μf*, then *P*_1_ is stable. And no Hopf bifurcation appears around the equilibrium *P*_1_.

**Proof**

It is easy to see Tr(*J*(*P*_1_)) = −(*σ*/*x*^*∗*^ + *fβy*^*∗*^) < 0. Noting *σβ* > *μf*, it is easy to see that

(19) det⁡JP1≥y∗x∗1+y∗2σβf−μ1+y∗2+α>0.

Thus the results are proved.

**Theorem 8 .**

Let (*x*^*∗*^, *y*^*∗*^) be the coordinate of the positive equilibrium *P*_1_. Then the following results hold:

1. If *fσβ* ≥ *μx*^*∗*2^, then *P*_1_ is stable.
2. If *fσβ* < *μx*^*∗*2^, then *P*_1_ is stable for $y^{*}>\sqrt{\alpha x^{*2}/(\mu x^{*2}-f\sigma \beta )}-1$ and unstable for $0<y^{*}<\sqrt{\alpha x^{*2}/\left(\mu x^{*2}-f\sigma \beta \right)}-1$.

**Proof**

It is easy to see

(20) $$\begin{array}{c} \det\left(\;J\left(\;P_{1}\;\right)\;\right)=\frac{y^{*}x^{*}}{{\left(1+y^{*}\right)}^{2}}\left[\;ay^{*2}+2ay^{*}+a+\alpha \;\right], \end{array}$$

where *a* = 1/*x*^*∗*2^(*σfβ* − *μx*^*∗*2^).

Let

(21) $$\begin{array}{c} h\left(\;y^{*}\;\right)=ay^{*2}+2ay^{*}+a+\alpha . \end{array}$$

Then the sign of the det⁡(*J*(*P*_1_)) is the same as that of *h*(*y*^*∗*^). Let Δ = 4*α*(*μ* − *σfβ*/*x*^*∗*2^). If *fσβ* = *μx*^*∗*2^ for any *y*^*∗*^ > 0, then *a* = Δ = 0. Hence,

(22) $$\begin{array}{c} h\left(\;y^{*}\;\right)=ay^{*2}+2ay^{*}+a+\alpha =\alpha >0. \end{array}$$

Then the first result can be obtained easily.

If *fσβ* > *μx*^*∗*2^, that is, *a* > 0, Δ < 0, then *h*(*y*^*∗*^) > 0 for any *y*^*∗*^ > 0. That is to say, *P*_1_ is stable as *fσβ* > *μx*^*∗*2^. On the other hand, one knows *a* < 0 as *fσβ* < *μx*^*∗*2^. Noting *α* > *μ*, then

(23) $$\begin{array}{c} a+\alpha =\frac{1}{x^{*2}}\left(\;\sigma f\beta -\mu x^{*2}+\alpha x^{*2}\;\right)>0. \end{array}$$

Then *h*(*y*^*∗*^) = 0 has roots $-\sqrt{\alpha x^{*2}/(\mu x^{*2}-f\sigma \beta )}-1<0$ and $\sqrt{\alpha x^{*2}/(\mu x^{*2}-f\sigma \beta )}-1>0$. From *y*^*∗*^ > 0, we have *h*(*y*^*∗*^) < 0 as $y^{*}>\sqrt{\alpha x^{*2}/(\mu x^{*2}-f\sigma \beta )}-1$ and *h*(*y*^*∗*^) > 0 as $0<y^{*}<\sqrt{\alpha x^{*2}/(\mu x^{*2}-f\sigma \beta )}-1$. That is to say, the second result holds.

In the following, we give some simulation results of the above results. We consider the system (5) and the parameters suggested by V. A. Kuznetsov et al. Then system (5) becomes

(24) dxdt=0.1181+1.131xtyt1+yt−0.00311xtyt−0.3743xt,dydt=1.636yt1−0.002yt−xtyt.

**Figure 1:**
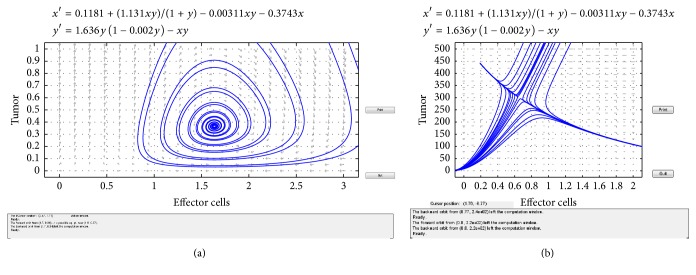
(a) Stable equilibrium *E*_1_. (b) Saddle-node equilibrium *E*_2_.

**Figure 2:**
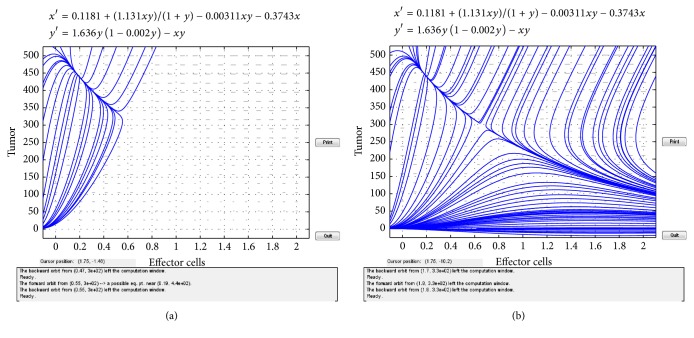
(a) Stable focus equilibrium *E*_3_. (b) Saddle node (*E*_2_) and stable focus (*E*_3_).

Obviously, system (24) has three positive equilibria: *E*_1_(1.6348,0.366269), *E*_2_(0.6538,300.188), and *E*_3_(0.1906,441.757). By simple computation, it is easy to know that the eigenvalues of *E*_1_ are −0.0367189 ± 0.599721*i*, and *E*_2_ has eigenvalues −1.45811 and 0.295245, and *E*_3_ has eigenvalues −1.68973 and −0.375447. These results are represented in Figures 1 and 2.

## 3. Dynamical Behaviors of the Model with Delay

In this section, we shall consider model (5) with delay

(25) dxdt=σ+αxy1+y−μxt−τyt−τ−δx,dydt=fy1−βy−xy.

The existence of the equilibria is the same as those of (5). The dynamical behaviors of the trivial equilibria and semitrivial equilibria are not difficult; we only study the positive equilibrium *P*_1_ = (*x*^*∗*^, *y*^*∗*^) here. The characteristic equation of linearized system for (25) at *P*_1_ takes the following form:

(26) $$\begin{array}{c} \lambda^{2}+B\lambda +C+\left(\;D\lambda +E\;\right)e^{-\lambda \tau }=0, \end{array}$$

where

(27) B=fβy∗−αy∗1+y∗+δ=fβy∗+fβy∗2+δ+y∗δ−α1+y∗,C=fβy∗2δ−α+fβy∗2δ−α+fβδ+αx∗1+y∗2,D=μy∗,E=μy∗2fβ−μx∗y∗=μfy∗2βy∗−1.

Similar to the proof in [7], then the following results can be obtained easily.

**Theorem 9 .**

(1) If

(28) B+D>0,C+E>0,D2−B2+2C<0,C2−E2>0  or  D2−B2+2C2<4C2−E2,

then all roots of (26) have negative real parts for all *τ* ≥ 0.

(2) If *C*^2^ − *E*^2^ < 0 or *D*^2^ − *B*^2^ + 2*C* > 0 and (*D*^2^ − *B*^2^ + 2*C*)^2^ = 4(*C*^2^ − *E*^2^), then (26) has a pair of purely imaginary roots ±*iω*_+_ at *τ* = *τ*_*j*_^+^.

(3) If *D*^2^ − *B*^2^ + 2*C* > 0, *C*^2^ − *E*^2^ > 0 and (*D*^2^ − *B*^2^ + 2*C*)^2^ > 4(*C*^2^ − *E*^2^), then (26) has a pair of purely imaginary roots ±*iω*_+_  (±*iω*_−_, resp.) at *τ* = *τ*_*j*_^+^  (*τ* = *τ*_*j*_^−^, *resp*.), where

(29) ω2±=12D2−B2+2C±D2−B2+2C2−4C2−E2,τj±=1ω±2jπ+arccos ω2−CE−DBω2E2+D2ω2,Dω2−CD+BE≥0,  j=1,2,…,1ω±2i+1π−arccos ω2−CE−DBω2E2+D2ω2,Dω2−CD+BE<0,  i=1,2,….

**Theorem 10 .**

(1) If the conditions of (1) in Theorem 9 hold, then *P*_1_ is asymptotically stable for any *τ* > 0.

(2) If *C*^2^ < *E*^2^ or the conditions of (3) in Theorem 9 hold, then *E*^*∗*^ undergoes Hopf bifurcation as *τ* = *τ*_0_.

(3) If *D*^2^ − *B*^2^ + 2*C* > 0, (*D*^2^ − *B*^2^ + 2*C*)^2^ = 4(*C*^2^ − *E*^2^) and *B*^2^ + 2*DτE* + *τ*^2^ > *D*^2^ + *E*^2^, then *E*^*∗*^ undergoes a Hopf bifurcation as *τ* = *τ*_0_.

**Proof**

From the analysis of the above, we only need to compute

(30) Sign dReλdτλ=iω=Sign±D2−B2+2C2−4C2−E2.

In third case, *d*(Re*λ*)/*dτ*|_*τ*=*τ*_0__ = 0, then we need the sign of the second derivative of Re*λ*(*τ*) of (26) at the point where *λ* is passing through *λ*_0_.

We can easily obtain

(31) $$\begin{array}{c} {Sign\left.\left(Re\lambda^{\prime \prime }\right)\right|}_{\lambda =i\omega }=\frac{\omega^{2}{\left(E^{2}+D^{2}\omega^{2}\right)}^{2}\left(E^{2}+D^{2}\omega^{2}\left(2\omega^{2}-2C-2D\tau E-\tau^{2}\right)-2E^{2}A-A^{2}\right)}{A^{3}} \end{array}$$

with

(32) $$\begin{array}{c} A=-B\omega^{2}-BC-DE+\tau \omega \left(\;E^{2}+D^{2}\omega^{2}\;\right). \end{array}$$

Since sgn⁡Re(*dλ*/*dτ*)^−1^|_*λ*=*iω*_ = 0, sgn⁡Im(*dλ*/*dτ*)^−1^|_*λ*=*iω*_ ≠ 0. Thus it is easy to see that *E*^2^ + *D*^2^*ω*^2^(2*ω*^2^ − 2*C* − 2*DτE* − *τ*^2^) − 2*E*^2^*A* − *A*^2^ < 0. This finishes the proof.

In the above, we obtained the conditions under which a family of periodic solutions bifurcated from the positive equilibrium at *τ* = *τ*_0_. In the following, we derive the explicit formulae for determining the properties of the Hopf bifurcated solution by using the normal form and the center manifold theory. Throughout this section, we always assume that the system (25) undergoes Hopf bifurcations at the positive equilibrium *P*_1_(*x*^*∗*^, *y*^*∗*^) for *τ* = *τ*_0_ and ±*iω* are the corresponding pure imaginary roots.

Let *t* = *τt*′, *x*(*t*) = *x*(*τt*′). Then (25) becomes

(33) dxdt=τσ+αxy1+y−μxt−1yt−1−δx,dydt=τfy1−βy−xy.

Set *τ* = *τ*_0_ + *p*, *p* ∈ *R*. Then *p* = 0 is a Hopf bifurcation value for (33). Equation (5) can be written as

(34) $$\begin{array}{c} x^{\prime }\left(\;t\;\right)=L_{p}\left(\;x_{t}\;\right)+F\left(\;p,x_{t}\;\right), \end{array}$$

where *x*(*t*) = (*x*_1_(*t*), *x*_2_(*t*))^*T*^ ∈ *R*^2^, and *L*_*p*_ : *C* → *R*, *F* : *R* × *C* → *R* are given, respectively, by

(35) Lpϕ=A1ϕ10ϕ20+A2ϕ1−1ϕ2−1,Fp,ϕ=τ0+p−αx∗1+y∗3ϕ220+α21+y∗2ϕ10ϕ20−μϕ1−1ϕ2−1−fβϕ220−ϕ10ϕ20,

with *ϕ*(0) = (*ϕ*_1_(0), *ϕ*_2_(0))^*T*^ ∈ *C* and

(36) A1=τ0+pαy∗1+y∗−δαx∗1+y∗2−y∗−fβy∗,A2=τ0+p−μy∗−μx∗00.

By Riesz representation theorem, there exists a function *η*(*θ*, *p*) of bounded variation for *θ* ∈ [−1,0] such that

(37) $$\begin{array}{c} L_{p}\left(\;\theta \;\right)=\overset{0}{\underset{-1}{\int }}d\eta \left(\;\theta ,p\;\right)\phi \left(\;\theta \;\right). \end{array}$$

For *ϕ* ∈ *C*^1^([−1,0], *R*^3^), define

(38) Apϕ=dϕθdθ,θ∈−1,0,∫−10dηs,pϕs,θ=0,Rpϕ=0,θ∈−1,0,Fp,ϕ,θ=0.

Then system (34) is equivalent to

(39) $$\begin{array}{c} x_{t}^{\prime }=A\left(\;p\;\right)\left(\;x_{t}\;\right)+R\left(\;p\;\right)x_{t}, \end{array}$$

where *x*_*t*_(*θ*) = *x*(*t* + *θ*) for *θ* ∈ [−1,0]. For Ψ ∈ *C*^1^([0,1], (*R*^3^)^*∗*^), define

(40) $$\begin{array}{c} A^{*}\psi \left(\;s\;\right)=\left\{\begin{array}{cc} \frac{d\psi \left(s\right)}{ds}, & s\in \left(0,1\right],\\ \overset{-}{\underset{}{\int }}1_{0}d\eta^{T}\left(t,0\right)\psi \left(-t\right), & s=0, \end{array}\right. \end{array}$$

and a bilinear inner product

(41) ψs,ϕθ=ψ¯0ϕ0−∫−10∫ξ=0θψ¯ξ−θdηθϕξdξ,

where *η*(*θ*) = *η*(*θ*, 0). Then *A*(0) and *A*^*∗*^ are joint operators.

Suppose that *q*(*θ*) = (1, *ρ*)^*T*^*e*^*iθω*_0_*τ*_0_^ is the eigenvector of *A*(*θ*) corresponding to *iω*_0_*τ*_0_, with *ρ* = −*y*^*∗*^/(*fβy*^*∗*^ + *iω*_0_*τ*_0_). Then *q*^*∗*^(*s*) = *D*(1, *ρ*^*∗*^)*e*^*isω*_0_*τ*_0_^ is the eigenvector of *A*^*∗*^ with *ρ*^*∗*^ = (1/*y*^*∗*^)[*αy*^*∗*^/(1 + *y*^*∗*^) − *δ* − *μy*^*∗*^*e*^*iω*_0_*τ*_0_^ − *iω*_0_*τ*_0_], $D=1/(1+\bar{\rho }\rho^{*}+\tau \left(-\mu y^{*}-\mu x^{*}\bar{\rho }\right)e^{-i\omega_{0}\tau_{0}})$ such that 〈*q*^*∗*^(*s*), *q*(*θ*)〉 = 1, $\langle q^{*}(s),\bar{q}(\theta )\rangle =0$.

In the following, we use the ideas in Adam and Bellomo [1] to compute the coordinates describing center manifold *C*_0_ at *p* = 0. Define

(42) zt=q∗,xt,Wt,θ=xtθ−2Reztqθ.

On the center manifold *C*_0_, we have

(43) Wt,θ=WZt,z¯t,θ=W20θz22+W11θzz¯+W02θz¯22+⋯,

where *z* and $\bar{z}$ are local coordinates for the center manifold *C*_0_ in the direction of *q*^*∗*^ and ${\bar{q}}^{*}.$ Note that *W* is real if *x*_*t*_ is real. For *x*_*t*_ ∈ *C*_0_ of (39), we have

(44) zt=iω0τ0z+q¯∗0F0,Wz,z¯+2Rezqθ=iω0τ0z+gz,z¯,

where

(45) gz,z¯=q¯∗0F0Zt,z¯t=g20θz22+g11θzz¯+g02θz¯22+g21θz2z¯2+⋯.

From (43) and (44), we have

(46) $$\begin{array}{c} x_{t}=\left(\;x_{1t}\left(\;\theta \;\right),x_{2t}\left(\;\theta \;\right)\;\right)=W\left(\;t,\theta \;\right)+zq\left(\;\theta \;\right)+\bar{zq\left(\theta \right)} \end{array}$$

and *q*(*θ*) = (1, *ρ*)^*T*^*e*^*iθω*_0_*τ*_0_^. Comparing the coefficients of the above equality with (45), we obtain

(47) g20=2τD¯αx∗1+y∗3ρ2+α21+y∗2ρ−μρe−2iω0τ0−fβρ2−ρ,g11=2τD¯αx∗1+y∗3ρρ¯+α21+y∗22Reρ−μ2Reρ−fβρρ¯−2Reρ,g02=2τD¯αx∗1+y∗3ρ¯2+α21+y∗2ρ¯−μρ¯e2iω0τ0−fβρ¯2−ρ¯+αx∗1+y∗3W1120ρ+z2z¯α21+y∗2W2010ρ¯+W2020+W1110ρ+W1120+αx∗1+y∗3W2020ρ¯,g21=2τD¯αx∗W2020ρ¯+W1120ρ1+y∗3+αW2010ρ¯+W2020+W1110ρ+W112021+y∗2+μW201−1ρe−2iω0τ0+W202−1e−2iω0τ0+W111−1ρe−2iω0τ0+W112−1e−2iω0τ0−fβW2020ρ¯+W1120ρ−W2010ρ¯+W2020+W1110ρ+W1120.

In order to determine *g*_21_, we also need to compute *W*_20_(*θ*) and *W*_11_(*θ*) as follows:

(48) W˙=x˙t−z˙q−zq¯˙=AW−2Req∗¯0F0qθ,θ∈−1,0,AW−2Req∗¯0F0qθ+F0,θ=0,

(49) =AW+Hz,z¯,θ,

where

(50) Hz,z¯,θ=H20θz22+H11θzz¯+H02θz¯22+⋯.

On the center manifold *C*_0_ near the origin,

(51) $$\begin{array}{c} \dot{W}=W_{z}\dot{z}+W_{\bar{z}}\dot{\bar{z}}. \end{array}$$

Comparing the coefficients of *z*, we obtain

(52) A−2iωτW20θ=−H20θ,AW11=−H11θ,H20θ=−g20qθ−g¯02θ¯,H11θ=−g11qθ−g¯11q¯θ.

From (52) and the definition of *A*, one has

(53) W˙20θ=2iω0τ0W20θ+g20q0eiω0τ0θ+g¯02q¯0e−iω0τ0θ.

Solving the above ODE, then

(54) W20θ=−ig20ω0τ0q0eiω0τ0θ−ig¯023ω0τ0q¯0e−iω0τ0θ+E1e2iω0τ0θ,

where *E*_1_ = (*E*_1_^(1)^, *E*_1_^(2)^) ∈ *R*^2^ is a constant vector. Similarly, we obtain

(55) $$\begin{array}{c} {\dot{W}}_{11}=\frac{ig_{11}}{\omega_{0}\tau_{0}}q\left(\;0\;\right)e^{i\omega_{0}\tau_{0}\theta }-\frac{i{\bar{g}}_{11}}{\omega_{0}\tau_{0}}\bar{q}\left(\;0\;\right)e^{-i\omega_{0}\tau_{0}\theta }+E_{2}, \end{array}$$

where *E*_2_ = (*E*_2_^(1)^, *E*_2_^(2)^) ∈ *R*^2^ is a constant vector.

From the definition of *A* and the above equations, we can obtain

(56) E1=2Aαx∗1+y∗3ρ2+α21+y∗2ρ−μρe−2iω0τ00−fβρ¯2−ρ¯,

with

(57) A=2iω0+αy∗1+y∗−d−μy∗e2iω0τ0αx∗1+y∗2−μxe2iω0τ0−y∗2iω0−fβy∗−1.

In the same way, it is easy to get

(58) E2=2αy∗1+y∗−d−μy∗αx∗1+y∗2−μx−y∗2iω0−fβy∗−1·αx∗1+y∗3ρρ¯+α21+y∗22Reρ−μ2Reρfβρ¯2−ρ¯.

In order to determine *E*_1_ and *E*_2_, we can calculate *W*_20_(*θ*) and *W*_11_(*θ*) from (54) and (55). Furthermore, we can also determine *g*_21_. Thus we can be able to compute the following values:

(59) c10=i2ω0τ0g20g11−2g112−g0223+g212,μ2=−Rec10Reλ′τ0,β2=2Rec10,T2=−Imc10+μ2Imλ′τ0ω0τ0.

**Theorem 11 .**

Equation (25) undergoes Hopf bifurcation as *τ* = *τ*_0_. If *μ*_2_ < 0 (*μ*_2_ > 0), then the bifurcated periodic solution is supercritical (subcritical); if *β* < 0 (*β* < 0), then the bifurcating solution is stable (unstable). The period of the bifurcated periodic solution is *T*_2_.

In the following, we present some numerical results of system (25) at different values of *τ*. We choose the parameters the same as before; the system (25) becomes

(60) dxdt=0.1181+1.131xy1+y−0.00311xt−τyt−τ−0.3743x,dydt=1.636y1−0.002y−xy.

**Figure 3:**
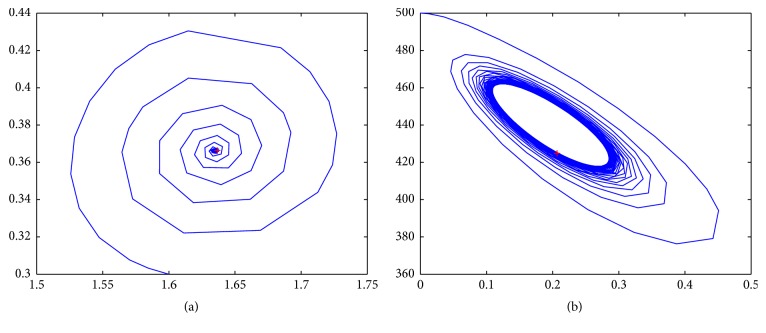
(a) Stable equilibrium *E*_1_ as *τ* = 1. (b) Bifurcated solution around *E*_3_ as *τ* = 0.7.

Obviously, system (60) has three positive equilibria: *E*_1_(1.6348,0.366269), *E*_2_(0.6538,300.188), and *E*_3_(0.1906,441.757). Equilibrium *E*_1_ is stable for all *τ*, while equilibrium *E*_2_ is unstable saddle node for all *τ*. When *τ* = 0, the positive equilibrium *E*_3_ is a stable focus, as *τ* is growing to *τ*_0_ = 0.707732, *λ*′(*τ*_0_) = 0.604929 − 0.176726*i*. When *τ* passes the critical value *τ*_0_, *E*_3_ loses its stability and a Hopf bifurcation occurs; that is, a family of periodic solutions bifurcate from *E*_1_. This is clearly demonstrated by Figure 3.

## 4. Conclusions

In 1994, Kuznetsov et al. [11] introduced a model, which describes competition between the tumor and immune cells. This mode describes the response of effector cells to the growth of tumor cells. Later, the model is simplified to a two-dimensional differential equation with Lotka-Volterra form immune response function *F*(*E*, *T*) = *θET*; the rich dynamical behaviors are studied such as stability, qualitative behaviors, and bifurcation behaviors.

In this paper, we studied the model with an immune response function with Michaelis-Menten form *F*(*E*, *T*) = *pET*/(*g* + *T*). In this case, the properties of system (4) are more complicated. There are so many tedious computations even for the existence of the equilibria. The dynamical behaviors of the model are more rich.

We have studied the nonlinear dynamics of a two-dimensional general differential system. We first provided linear analysis of the system at the possible equilibria, namely, the semitrivial and positive equilibria, and discussed the existence of Hopf bifurcation at the positive equilibrium. Then we consider the system with delay; we investigated the Hopf bifurcation of the system. The existence and stability of periodic solutions were given. Numerical simulations were presented to illustrate the theoretical analysis and results. Cancer immunosurveillance functions are taken as an important defense to cancer; this is the elimination process. In fact, the existence and stability of the semitrivial equilibrium correspond to the elimination process. Our results on the existence and stability of the Hopf bifurcated periodic solutions describe the equilibrium process. If a stable periodic orbit exists, then the tumor and the immune system can coexist for a long time, although the cancer cannot be eliminated. Furthermore, the parameters are important in controlling the development and progression of the tumor, which is decided by the conditions of the existence of the bifurcations.

The existence of oscillatory solutions in the tummor and immune system interaction models demonstrates that the phenomenon has been observed in some related models [7, 10, 13–15]. The initial values and delay are also important in the oscillatory coexistence of the tumor cells and the effector cells. Numerical simulations indicate this information well.
